# Inter-scanner reproducibility of brain volumetry: influence of automated brain segmentation software

**DOI:** 10.1186/s12868-020-00585-1

**Published:** 2020-09-04

**Authors:** Sirui Liu, Bo Hou, Yiwei Zhang, Tianye Lin, Xiaoyuan Fan, Hui You, Feng Feng

**Affiliations:** Department of Radiology, Peking Union Medical College Hospital, Chinese Academy of Medical Sciences and Peking Union Medical College, Beijing, 100730 China

**Keywords:** Magnetic resonance imaging, Automated brain volumetry, Coefficient of variation, Inter-scanner reproducibility

## Abstract

**Background:**

The inter-scanner reproducibility of brain volumetry is important in multi-site neuroimaging studies, where the reliability of automated brain segmentation (ABS) tools plays an important role. This study aimed to evaluate the influence of ABS tools on the consistency and reproducibility of the quantified brain volumetry from different scanners.

**Methods:**

We included fifteen healthy volunteers who were scanned with 3D isotropic brain T1-weighted sequence on three different 3.0 Tesla MRI scanners (GE, Siemens and Philips). For each individual, the time span between image acquisitions on different scanners was limited to 1 h. All the T1-weighted images were processed with FreeSurfer v6.0, FSL v5.0 and AccuBrain^®^ with default settings to obtain volumetry of brain tissues (e.g. gray matter) and substructures (e.g. basal ganglia structures) if available. Coefficient of variation (CV) was calculated to test inter-scanner variability in brain volumetry of various structures as quantified by these ABS tools.

**Results:**

The mean inter-scanner CV values per brain structure among three MRI scanners ranged from 6.946 to 12.29% (mean, 9.577%) for FreeSurfer, 7.245 to 20.98% (mean, 12.60%) for FSL and 1.348 to 8.800% (mean value, 3.546%) for AccuBrain^®^. In addition, AccuBrain^®^ and FreeSurfer achieved the lowest mean values of region-specific CV between GE and Siemens scanners (from 0.818 to 5.958% for AccuBrain^®^, and from 0.903 to 7.977% for FreeSurfer), while FSL-FIRST had the lowest mean values of region-specific CV between GE and Philips scanners (from 2.603 to 16.310%). AccuBrain^®^ also had the lowest mean values of region-specific CV between Siemens and Philips scanners (from 1.138 to 6.615%).

**Conclusion:**

There is a large discrepancy in the inter-scanner reproducibility of brain volumetry when using different processing software. Image acquisition protocols and selection of ABS tool for brain volumetry quantification have impact on the robustness of results in multi-site studies.

## Background

Reproducible in vivo segmentation and qualification of brain tissues in toto (e.g. white matter (WM), gray matter (GM), cerebrospinal fluid (CSF)) and specific substructures (e.g. hippocampus and thalamus) are of vital importance to facilitate clinic decisions of diseases related to brain morphometry [1]. Brain segmentation methods include manual segmentation, semiautomatic segmentation and automatic brain segmentation (ABS) [2]. Both manual and semiautomatic segmentations require manual delineation of brain regions, which are unavoidably susceptible to intra- and inter-rater inconsistency [2, 3]. In contrast, ABS is hand-free and thus more resistant to inter-rater variability. Regarding the diseases related to abnormal brain morphometry, it provides a more effective and objective pipeline to yield reproducible quantifications of brain volumetry, which can facilitate to make accurate diagnosis, monitor disease progression and evaluate the prognosis [1].

In the recent decade, there have been dramatically more and more multi-site clinical studies as it becomes easier to obtain large data from multiple partners worldwide regarding the patient population in question [4]. In such background, the time-saving and objective ABS tools play a key role in large-scale multi-site brain morphometry studies based on MR images [5]. In fact, the accuracy and reproducibility of ABS tools (i.e. segmentation software) can greatly affect the evaluation of subtle brain morphometry changes [6]. It is not possible to make a correct diagnostic or treatment decision if the applied ABS tools produce inconsistent results of brain volumetry. Therefore, it is important to evaluate the variations of the quantified brain volumetry from different ABS software (for example, by testing their reproducibility on multiple scanners) before application in clinical practice.

To focus on the performance of ABS software and minimize the influences of other possible factors, some studies used standard datasets to evaluate the reproducibility of various image segmentation and volumetry software (e.g., SPM, FSL, Freesurfer) [2, 7]. However, in addition to segmentation methods, there are many other factors that affect the quantified brain volumetric measures, such as imaging parameters, scanner manufacturer, subject positioning and hydration status, as well as image artifacts [5, 8, 9]. The existing studies also suffer from limitations in different aspects, for example: (1) only a small number of brain structures are considered [10, 11]; (2) only one ABS software is tested without comparison of performance with other ABS software [1, 5]; and (3) only a small sample is used for performance evaluation which cannot exclude the effect of interactions between scanners and subjects [1, 12].

To this end, this study aimed to evaluate the inter-scanner reproducibility of brain volumetry quantified by different ABS software in a more comprehensive way that can be generalized to clinical practice. We compared three ABS software, i.e. Freesurfer [13], FIRST toolbox in FSL [14] and AccuBrain^®^ (BrainNow Medical Technology Ltd.) [15], in terms of their quantification performance in automatic brain volumetry. The accuracy and reliability of Freesurfer and FSL have been tested previously [1, 2, 6]. All the above segmentation tools can automatically segment and quantify multiple brain structures. FreeSurfer implements a complex image processing pipeline to segment a lot of anatomical structures and measure their volumes [13]. FIRST in FSL is a model-based segmentation tool that enables segmentations of fifteen subcortical structures, such as thalamus, caudate, putamen and so on. AccuBrain^®^ is a cloud-based tool of automated brain volumetry. In this study, we compared the coefficients of variation [1] of the quantified brain volumetry of these tools in inter-scanner acquisitions to test their reproducibility and reliability.

## Methods

### Subjects and imaging protocol

Fifteen healthy volunteers (5 males and 10 females, mean age: 25.1 ± 0.59 years old) were enrolled in this study. The inclusion criteria in our study were: (a) no medical history of central neural system disease or psychiatric disorder; (b) Mini-Mental State Examination (MMSE) score within the normal range (27–30); (c) normal in physical examination of the central nervous system; (d) no medical treatments that may result in brain volumetric changes (e.g. steroid treatment) during the whole period of MRI acquisitions.

All the subjects were scanned using 3D sagittal isotropic brain T1-weighted sequences on three different 3.0 Tesla (T) MRI scanners, including GE Discovery MR750, Siemens Skyra and Philips Ingenia CX within 1 day. To avoid time-related brain structural volume changes, the time span of acquiring T1-weighted images on three different MR scanners for each subject was limited to 1 h. The details about the MRI scanners and the imaging protocols as conventionally used in clinic [1, 16] are listed in Table 1.

**Table 1:** Imaging protocols of the tested MRI scanners

Imaging protocols	GE Discovery MR750	Siemens Skyra	Philips Ingenia CX
Field strength (T)	3.0	3.0	3.0
Sequence name	BRAVO	tfl3d1	T1W_3D_TFE
Sequence type	T1 inversion-prepared FSGRE	MPRAGE	3D TFE
TR (ms)	6.7	1900	8.2
TE (ms)	2.9	2.5	3.7
TI (ms)	400.0	900.0	964.9
FA (°)	12	9	8
Matrix	256 × 256	256 × 256	256 × 256
FOV (mm)	256 × 256	256 × 256	256 × 256
Slice thickness(mm)	1	1	1
Angulation	Sagittal	Sagittal	Sagittal
Voxel size (mm)	1 × 1	1 × 1	1 × 1
Number of slices	190	192	190
TA (min:s)	05:51	04:31	05:34

*FA* flip angle, *FOV* field of view, *TA* acquisition time, *TE* echo time, *TI* inversion time, *TR* repetition time

### Image processing

Visual assessment was performed on the obtained T1-weighted scans to confirm that there were no severe common artifacts (e.g. motion artifact and metal artifact), brain lesions or brain atrophy, which may lead to inaccurate volumetric estimations from the images. Subsequently, all the 3D T1-weighted MR images were processed using FreeSurfer v6.0, FSL v5.0 and AccuBrain^®^.

1. FreeSurfer (http://surfer.nmr.mgh.harvard.edu/) is an atlas-based open-source software for processing and analyzing structural brain MRI images with no human intervention. The atlas that contains brain anatomy information is used as a reference for the segmentation of new MRI images [3]. Labels of brain regions from the atlas are modulated by affine transformations to fit target images [2]. FreeSurfer encompasses template registration and segmentation, and it can measure not only the volumes of many anatomical structures [13] but also other brain structural features such as cortical thickness, surface area, intensity and curvature. In this study, the images were processed using “recon-all” script provided by FreeSurfer, and a summary of volumetry of multiple brain structures were calculated.
2. FIRST (https://fsl.fmrib.ox.ac.uk/fsl/fslwiki/FIRST) is provided as part of the FSL software distribution. It is a model-based segmentation tool. The models are created from manually labelled and segmented MRI images which are offered by the Center for Morphometric Analysis. These labels are parameterized as surface meshes and modelled as a point distribution model. Here, we used the “run_first_all” command of FSL-FIRST to calculate the brain volumetry of the provided fifteen subcortical structures.
3. AccuBrain^®^ is a cloud-based tool for automatic brain quantification [15]. After uploading the DICOM files on the website, a report including brain volumetry and a summary of anatomy information will be provided. AccuBrain^®^ employs multi-atlas image registration-based segmentation procedure. It uses a large atlas pool which is consisted of hundreds of brain MR images obtained from different scanners. Based on similarity measures, it selects a batch of most similar brains from the atlas pool to segment the subject image.

To perform a fair comparison of the quantification results in a way as similar as in clinical practice, we used the default settings of all these tools without any specific preference in parameter selection [2].

### Reproducibility analysis

**Fig. 1:**
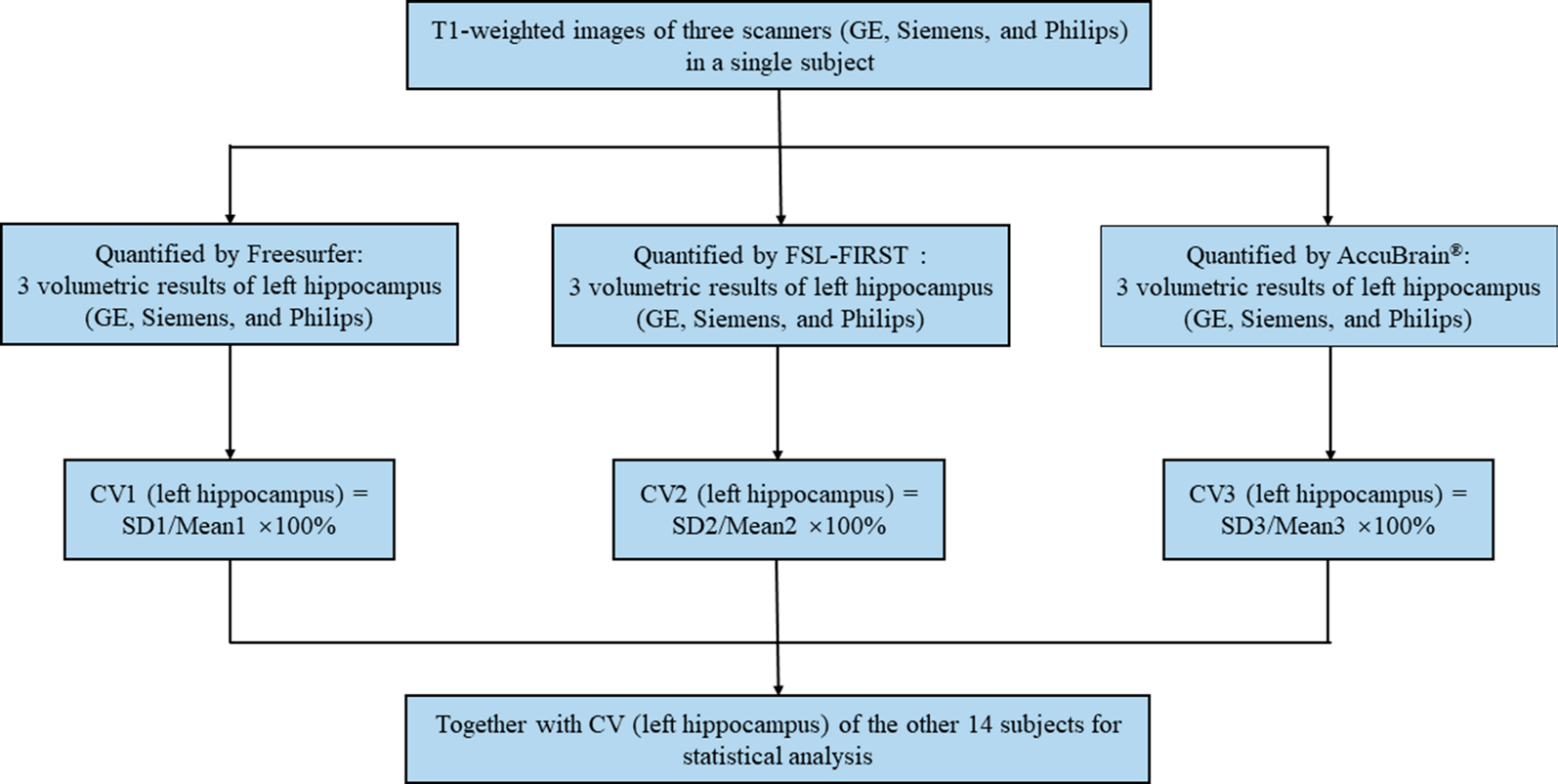
Analysis method of coefficient of variation of left hippocampus. CV: coefficient of variance; SD: standard deviation of volumetric results of left hippocampus from three scanners; Mean: arithmetic mean of volumetric results of left hippocampus from three scanners

In order to test inter-scanner variability of brain volumetry, we measured the coefficient of variation (CV) of the quantified volumetric data based on the MRI acquisitions from different scanners. With a specific quantification tool for a certain brain region, the CV value was first calculated for each subject to measure the variability of brain regional volumes from acquisitions of the three scanners (GE, SIEMENS and PHILIPS). In detail, it is calculated as the proportion of standard deviation (SD) to the mean of volumetric measures from different scanners, which can also be expressed as a formula: ${\text{CV}} = \sigma /m \times 100\%$, where $\sigma$ is the standard deviation and *m* is the arithmetic mean of the region-specific volumetric results of a single subject among different acquisitions. For example, if we would like to quantify the inter-scanner variability of the volumetric data of left hippocampus as measured by FreeSurfer (Additional file 1) for a single subject, we need to calculate the mean and SD of the three quantification results (from three scanners respectively) and subsequently the CV (i.e. SD/mean). In this way, we got a CV of the three scanners when quantifying left hippocampus with FreeSurfer. Similarly, we can calculate the CV of left hippocampus volume for this subject when using FSL-FIRST or AccuBrain^®^ for quantification. Finally, the CV values obtained from specific quantification tools can be compared in a cohort-level and for the volumetric measures of other brain substructures. Figure 1 is the flow chart of analysis method of CV of left hippocampus.

Due to the limited sample size, we utilized a non-parametric test, i.e. the Wilcoxon signed-rank test, to investigate the pair-wise between-group differences regarding the CV values of different ABS tools.

## Results

**Fig. 2:**
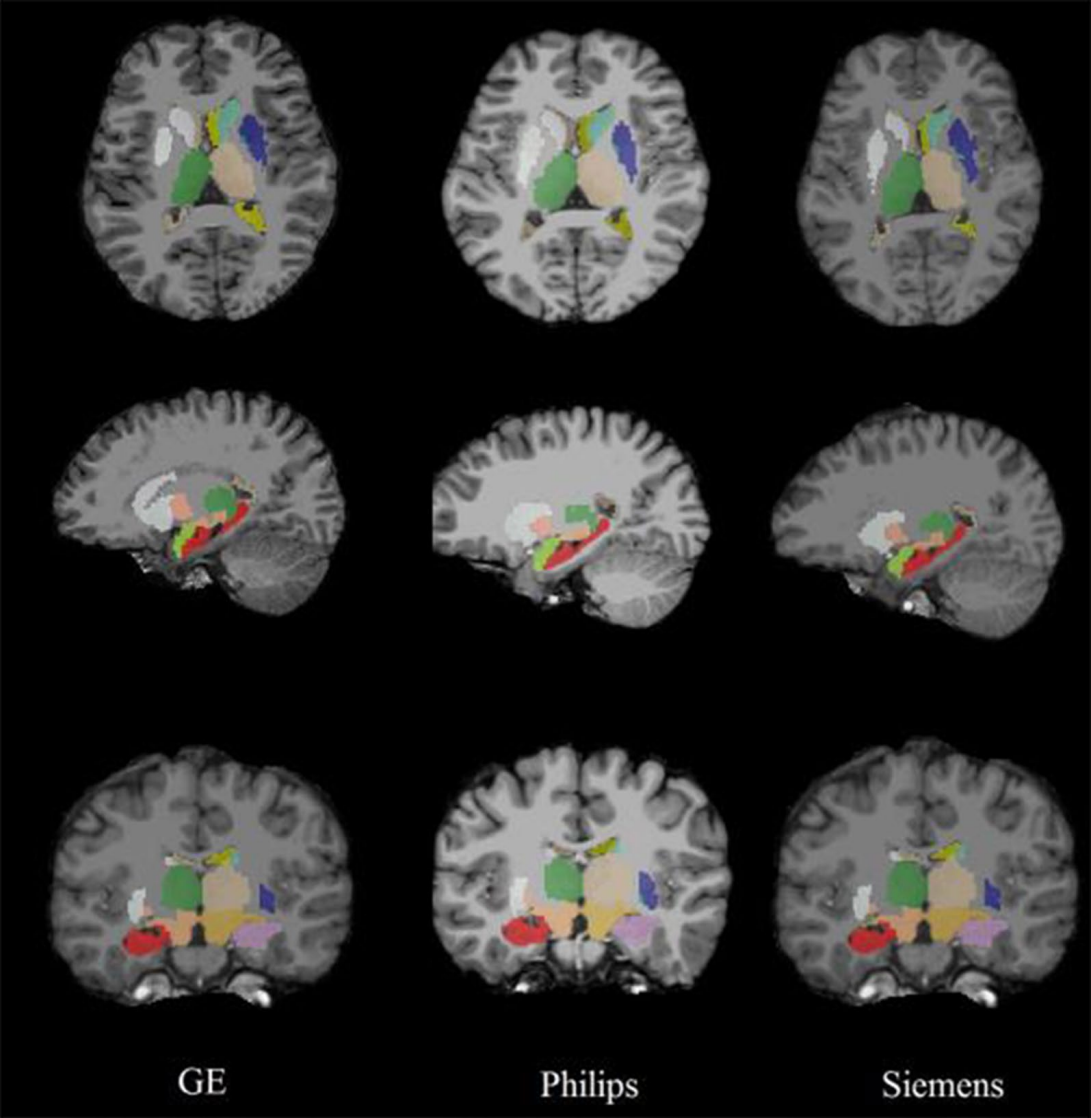
FreeSurfer segmentation results of different MRI acquisitions

**Fig. 3:**
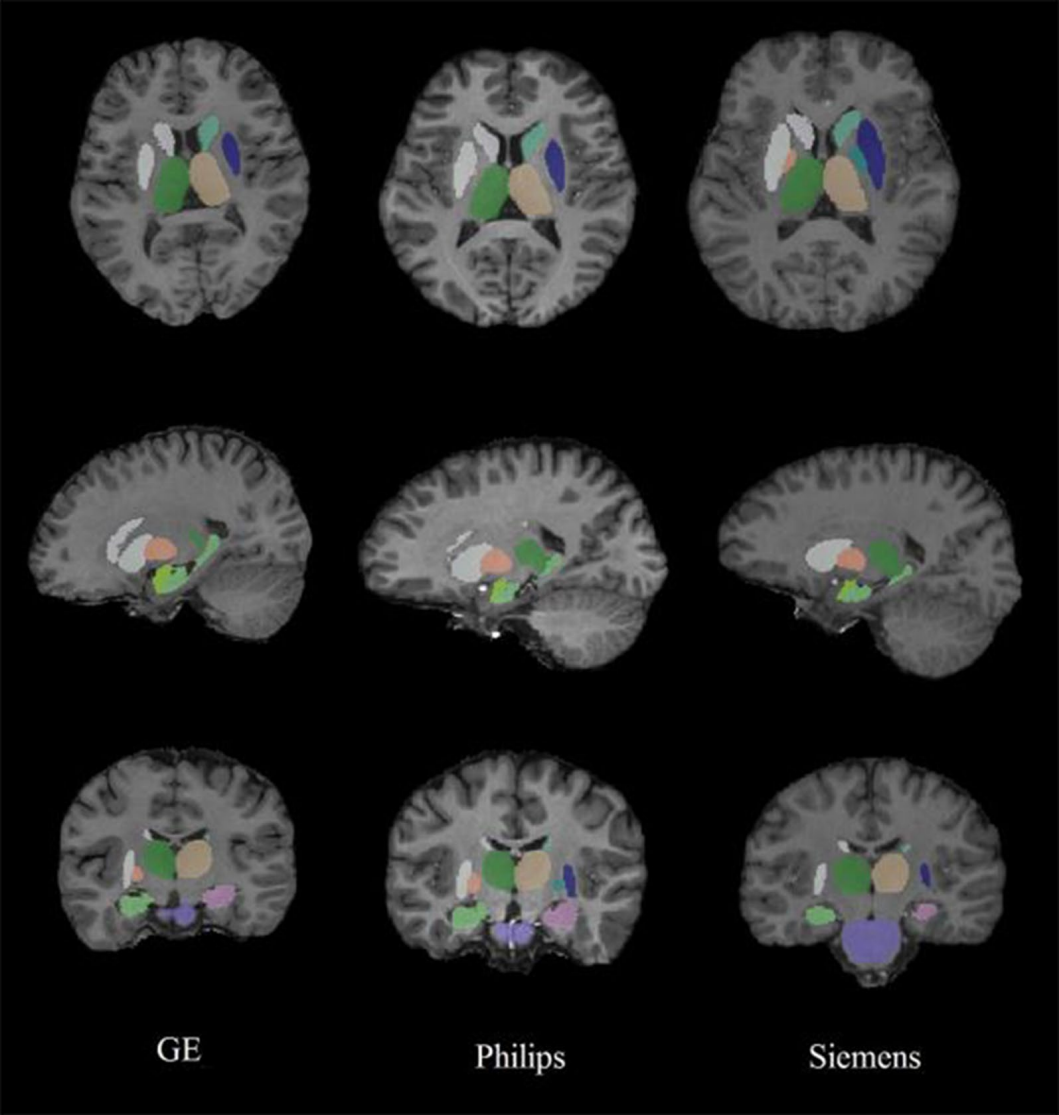
FSL-FIRST segmentation results of different MRI acquisitions

**Fig. 4:**
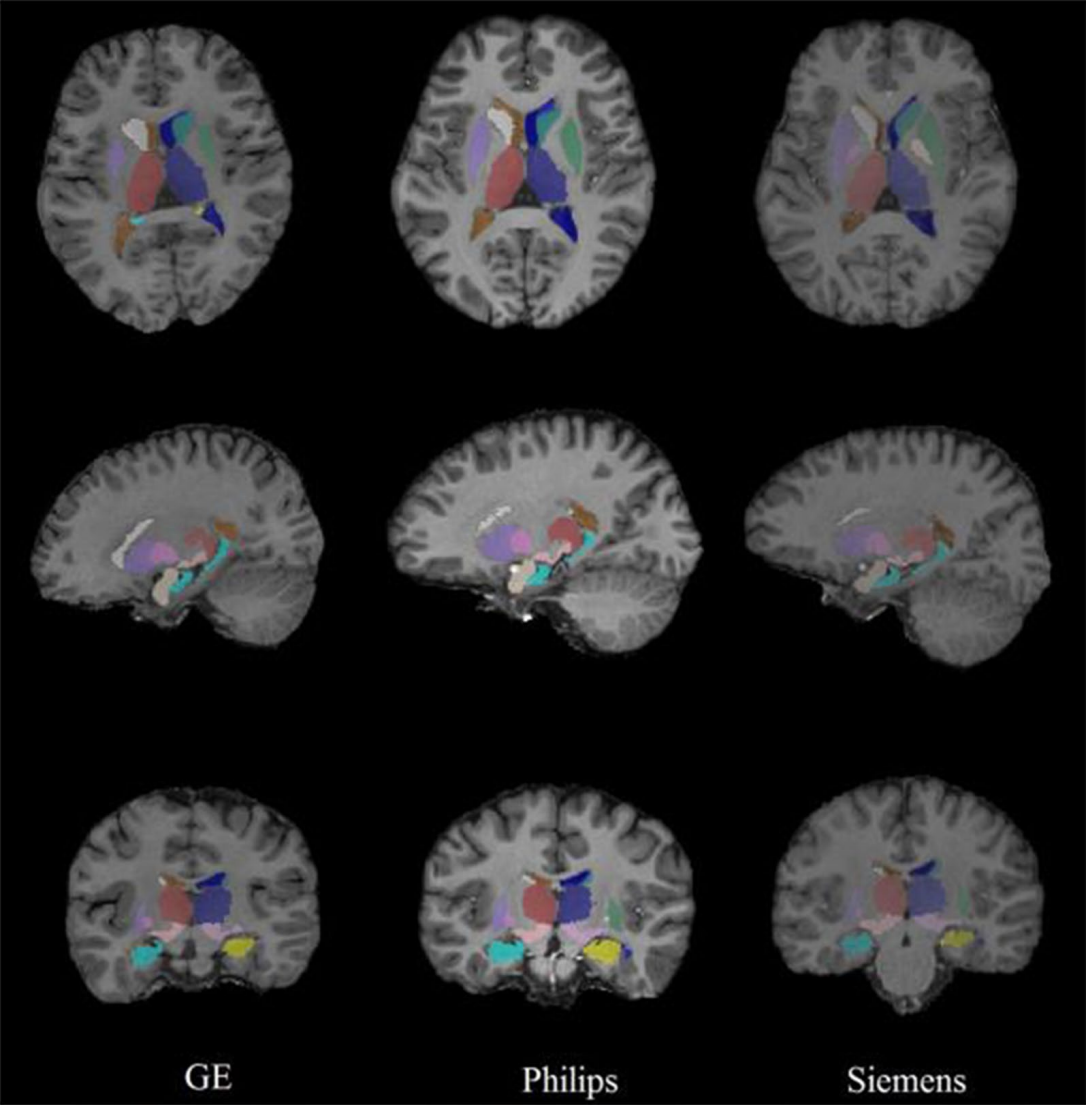
AccuBrain^®^ segmentation results of different MRI acquisitions

Figures 2, 3 and 4 show some segmentation results by FreeSurfer (Fig. 2), FSL-FIRST (Fig. 3) and AccuBrain^®^ (Fig. 4), from which we can visually compare the segmentation quality.

The quantified brain structures with their volumetric measures from different ABS tools were listed in Additional file 2 for reference. Of note, FSL-FIRST only quantified subcortical regions and thus the volumetric measures of WM, GM, and ventricular structures (e.g. lateral ventricle) were not available in FSL-FIRST. The CV values of the brain volumetric measures quantified from different ABS tools and the pair-wise comparisons of the CV values among these software were shown in Table 2.

**Table 2:** Coefficient of variation (CV) for inter-scanner volumetric measurements among GE, Siemens and Philips

	Coefficient of variation			p-value		
	FreeSurfer	FSL-FIRST	AccuBrain	FreeSurfer vs. FSL-FIRST	AccuBrain vs. FreeSurfer	AccuBrain vs. FSL-FIRST
WM	8.117	N.A.	1.348	N.A.	0.005	N.A.
GM	6.946	N.A.	1.927	N.A.	< 0.001	N.A.
Hippocampus L	7.706	9.600	8.800	0.151	0.978	0.679
Hippocampus R	8.459	10.57	8.144	0.489	0.561	0.064
Amygdala L	10.35	17.15	3.768	0.010	< 0.001	< 0.001
Amygdala R	10.23	20.98	3.557	0.004	0.002	< 0.001
Lateral ventricle L	9.123	N.A.	2.547	N.A.	0.001	N.A.
Lateral ventricle R	8.631	N.A.	2.892	N.A.	0.008	N.A.
VentralDC L	9.809	N.A.	4.298	N.A.	0.018	N.A.
VentralDC R	9.350	N.A.	3.685	N.A.	0.022	N.A.
Thalamus L	8.632	8.919	1.635	0.679	< 0.001	< 0.001
Thalamus R	8.440	9.739	1.811	0.524	< 0.001	< 0.001
Caudate L	9.597	12.91	2.750	0.083	< 0.001	< 0.001
Caudate R	9.331	14.11	2.314	0.073	0.001	< 0.001
Putamen L	10.77	11.61	2.755	0.720	< 0.001	< 0.001
Putamen R	9.793	11.64	2.471	0.330	0.001	< 0.001
Pallidum L	12.29	7.245	4.025	0.018	< 0.001	0.007
Pallidum R	11.77	9.306	2.316	0.389	0.001	< 0.001
Accumbens L	11.72	13.93	4.859	0.679	< 0.001	0.001
Accumbens R	10.43	18.75	5.014	0.015	0.002	< 0.001
Average	9.577	12.60^a^	3.546	0.041	0.001	< 0.001

The mean values of region-specific CV (i.e. inter-scanner variability in brain volumetry of the three scanners) of the examined 15 subjects are displayed for each ABS tool, and the associated pairwise comparison results (i.e. p values) of the coefficients of variation among the ABS tools are also provided

*GM* gray matter, *N.A*. not available, *WM* white matter, *VentralDC* ventral diencephalon, *L* Left, *R* Right

The average CV of FSL-FIRST for different brain substructures is the mean over the structures available for quantification in FSL-FIRST (i.e. hippocampus, amygdala, thalamus, caudate, putamen, pallidum and accumbens)

The mean inter-scanner CV values among three different MRI scanners ranged from 6.946% (GM) to 12.29% (right pallidum) with a mean value of 9.577% for FreeSurfer, and 7.245% (left-pallidum) to 20.98% (right-amygdala) with a mean value of 12.60% for FSL-FIRST. In comparison, the CV values of AccuBrain^®^ were much smaller, ranging from 1.348% (WM) to 8.800% (left hippocampus) with a mean value of 3.546% (Table 2). Comparing FreeSurfer and FSL-FIRST, the CV values of different brain regions were generally similar, except for three regions where the FreeSurfer performed better (i.e. left and right amygdala, right accumbens, p < 0.05) and one region where FSL-FIRST performed better (i.e. left pallidum, p = 0.018). Regarding AccuBrain^®^, it achieved significantly smaller inter-scanner CV values than FSL-FIRST and FreeSurfer in almost all the regions that were tested, except for left and right hippocampus, where no significant difference of CV values was found among these three software.

We further investigated the inter-scanner variability in each pair of scanners (GE vs. Philips, GE vs. Siemens, Philips vs. Siemens) as shown in Table 3. When using FreeSurfer and AccuBrain^®^ for automated brain volumetry, the variability between GE and Siemens scanners was the least among the comparisons of all the tested regions. When applying FSL-FIRST for quantification, the inter-scanner variability between GE and Philips was the least. In addition, AccuBrain^®^ also achieved the lowest variability of brain volumentry between Siemens and Philips scanners compared to FreeSurfer and FSL-FIRST.

**Table 3:** Coefficient of variation (CV) for inter-scanner volumetric measurement between each pair of scanners

	Freesurfer			FSL-FIRST			AccuBrain		
	GE vs. PHILIPS	GE vs. SIEMENS	PHILIPS vs. SIEMENS	GE vs. PHILIPS	GE vs. SIEMENS	PHILIPS vs. SIEMENS	GE vs. PHILIPS	GE vs. SIEMENS	PHILIPS vs. SIEMENS
WM	8.393	0.903	7.874	N.A.	N.A.	N.A.	0.999	0.818	1.138
GM	6.835	1.142	7.223	N.A.	N.A.	N.A.	1.581	1.357	1.376
Hippocampus L	8.165	2.583	6.471	7.802	7.603	7.814	8.810	5.916	6.615
Hippocampus R	8.947	2.062	7.723	8.160	8.431	8.949	6.636	5.958	6.374
Amygdala L	9.192	7.257	7.429	15.23	8.490	18.15	2.029	3.246	3.233
Amygdala R	9.765	2.699	9.996	13.86	13.62	21.76	3.000	1.756	3.385
Lateral ventricle L	7.307	4.174	9.454	N.A.	N.A.	N.A.	2.332	1.352	2.419
Lateral ventricle R	8.131	2.478	8.356	N.A.	N.A.	N.A.	2.684	2.086	2.763
VentralDC L	9.536	2.908	9.250	N.A.	N.A.	N.A.	4.573	4.217	1.268
VentralDC R	9.621	2.952	8.060	N.A.	N.A.	N.A.	3.918	3.222	1.459
Thalamus L	8.538	2.611	7.701	3.052	8.805	8.357	1.569	0.984	1.354
Thalamus R	8.576	2.672	9.367	2.603	9.811	9.126	0.928	1.676	1.676
Caudate L	8.058	4.347	9.700	7.599	12.30	10.25	2.875	1.308	1.627
Caudate R	8.678	2.803	9.218	9.815	11.28	12.37	2.147	1.463	1.727
Putamen L	10.70	4.854	8.713	7.811	13.66	6.006	2.663	2.022	1.788
Putamen R	9.662	3.231	8.733	8.854	13.26	6.034	2.231	2.590	1.499
Pallidum L	9.381	7.032	12.41	5.857	7.079	4.253	4.174	3.178	2.232
Pallidum R	8.093	7.788	11.46	6.331	9.790	6.236	2.158	1.418	1.889
Accumbens L	9.714	7.977	9.544	7.800	10.96	13.44	3.572	3.583	4.223
Accumbens R	8.844	6.046	9.091	16.31	12.92	14.93	4.093	2.793	4.671
Average.	8.807	3.926	8.789	8.650	10.57	10.55	3.149	2.547	2.636

The mean values of region-specific CV (i.e. inter-scanner variability in brain volumetry of GE vs. PHILPS, GE vs. SIEMENS and PHILIPS vs. SIEMENS) of the examined 15 subjects are displayed for each ABS tool

*GM* gray matter, *N.A*: not available, *WM* white matter, *VentralDC* ventral diencephalon, *L* Left, *R* Right

The average CV of FSL-FIRST for different brain substructures is the mean over the structures available for quantification in FSL-FIRST (i.e. hippocampus, amygdala, thalamus, caudate, putamen, pallidum and accumbens)

## Discussion

In multi-site neuroimaging studies, it is important to examine the inter-scanner reproducibility of volumetry data acquired from different MRI scanners before further statistical analysis with the integrated data. To this aim, MRI images of fifteen healthy subjects acquired multiple times from different MRI scanners were collected for scanner-related comparison and three structural brain MRI analysis software (FreeSurfer, FSL-FIRST and AccuBrain^®^) were selected to test software-related differences in measurements of brain volumetry. The segmentation accuracies of the three software have been evaluated and compared in many literatures [13]. As the segmentation accuracy of different structures is highly dependent on the anatomical definition of structures in a specific software, the comprehensive comparison of region-specific segmentation accuracy among the different software is out of the scope of this study. Our major objective is to investigate the reproducibility of brain volumetry in inter-scanner acquisitions and to test the influence of quantification software selection on inter-scanner reproducibility of brain volumetry.

In this study, AccuBrain^®^ presented less inter-scanner variability than FreeSurfer and FSL-FIRST according to the comparison of their CV values of brain volumetry. These findings might result from the superior performance of AccuBrain^®^ due to its large atlas pool, which consists of template images from a wide range of MRI scanners for knowledge transfer. Although FreeSurfer also employs atlas-based segmentation, it uses only one specific atlas (including one MRI template with labeled atlas) for knowledge transfer, which may influence its performance in inter-scanner reproducibility. Furthermore, several brain substructures (e.g. hippocampus, amygdala, pallidum and accumbens) had relatively higher CVs than other structures in the tested ABS tools, while brain tissues with larger volume (e.g. WM and GM) presented much smaller CV values (Table 2). This finding may result from the relative volume of the tested brain structures or tissues, where the misclassified voxels from segmentation may have larger impact on the CV values if the volume of the structure is small. The secondary cause may be the differences in boundary definition and tissue contrast. One of the most important features that triggers brain MRI segmentation is brain tissue intensity [3, 15], and the fuzzy boundary and lower contrast of background are more likely to cause tissue misclassification.

In addition, we found that the variabilities of the quantified brain volumetry between each pair of scanners (GE vs. Philips, GE vs. Siemens, Philips vs. Siemens) were quite different when different ABS tools were used (Table 3). When using AccuBrain^®^ or FreeSurfer as the quantification tool, the inter-scanner variability of GE and Siemens scanners was the lowest compared with the other pairs of scanners, and when using FSL-FIRST, the inter-scanner variability between GE and Philips scanners was the lowest. In view of the segmentation algorithm, both AccuBrain^®^ and FreeSurfer employ atlas-based segmentation method, while FSL-FIRST uses model-based segmentation method. The performance of atlas-based segmentation depends on the matching of the intensity in template image and that in the image to be segmented, while model-based segmentation relies more on fitting a prior model for the image to be segmented. In fact, the images acquired from GE and Siemens scanners are more similar in terms of intensity level and image contrast than the other pairwise comparison of scanners, which may also serve as a reason for the better reproducibility of the data from GE and Siemens scanners with AccuBrain^®^ and FreeSurfer. In contrast, FSL-FIRST, which is less affected by intensity level, does not follow the similar trend of pairwise inter-scanner variability in brain volumetry as identified by AccuBrain^®^ and FreeSurfer. In fact, FSL-FIRST presented the highest CV values among all the pair-wise inter-scanner comparisons, indicating its inferior inter-scanner reproducibility. Regarding the applications of the three segmentation tools, they all have their own superiorities. For example, although FreeSurfer takes the longest time to process one dataset, it supports not only quantification of subcortical brain volumetry, but also cortical parcellation and quantification. FSL-FIRST tool also enables surface-based morphometry analysis for the subcortical structures in addition to quantification of brain volumetry. As this paper mainly discussed about the reproducibility of brain volumetric quantification as affected by ABS tools, the comparison regarding different functions of the mentioned ABS tools is out of the scope of this study.

Of note, if the CVs (that indicate inter-scanner variability in brain volumetric quantification) are relatively higher when involving comparisons with a specific scanner, it does not necessarily imply that this scanner is inferior to the others, as the contrast and intensity level can be changed by modulating imaging parameters [15]. Although segmentation algorithm is the primary factor that influences inter-scanner reproducibility, the effect of the pulse sequence selected for a specific scanner cannot be underestimated, since it also has a large impact on the quantification results of brain volumetry. The misclassification rates can be reduced by a suitable and proper choice of pulse sequences [17], and the CV values obtained in our study may be reduced by adjustments of image acquisition parameters, which warrants further validations in the future.

Segmentation and quantification of specific brain regions are common tasks in the study of neurological disorders such as movement disorders [18], Alzheimer’s disease [19] and epilepsy [20]. Disease progression is often reported using annualized rate of tissue volume loss, which may be very small [2]. Therefore, highly reproducible measurements are important to detect and monitor brain volumetric changes at multiple time points. Routine use of brain morphology analysis in clinical nursing needs reliable and reproducible measurements, because radiologists often give advice on treatment decisions according to brain volumetric changes [2]. High reproducibility is also necessary for detecting the subtle yet important changes of brain disease, especially in multi-site researches. The change of interest cannot be studied if the inter-scanner reproducibility of brain volume has large discrepancy [21, 22]. In such background, the proper selection of brain segmentation software is a critical step in computer-aided diagnosis and measurement [3]. In addition, choosing same scanner manufacturer, field strength, head coil, magnetic gradient [23], and pulse sequence [9] is helpful to improve inter-scanner reproducibility.

There are some limitations of this study that need to be considered. First, the results of our study were grounded on the examinations of young healthy volunteers. Therefore, the variability of brain volumetry in a cohort with severe brain atrophy and/or with brain lesions remain unclear. The accuracy of ABS tools might decrease when brain anatomic segmentation is performed in patients with demyelinating lesions (e.g. multiple sclerosis), mass-like lesions (e.g. tumors) [24] or brain atrophy. In this respect, further studies with focus on the reproducibility of ABS tools in brain volumetry should expand the cohort to be tested from healthy individuals to individuals with brain lesions and/or atrophy. Second, as the primary goal of this study was to test inter-scanner reproducibility in a way as in clinical practice, the applied imaging parameters in this study were all daily used in clinic without any additional modulation, and the software parameters were set as default without specific preference in parameter selection [2]. However, it has been reported that appropriate adjustments of image acquisition parameters can help achieve better reproducibility of brain volumetry [25]. Therefore, future efforts should also aim to investigate the optimal imaging parameters and protocols to further improve the inter-scanner reproducibility in multicenter studies.

## Conclusion

In conclusion, this study demonstrated that automatic brain segmentation tool has a considerable impact on the inter-scanner reproducibility in quantification of brain volumetry. The results of this study may facilitate neuroimage data sharing and integration in multi-site research, where the selection of an appropriate automated brain quantification tool serve as a prerequisite to obtain reliable and meaningful findings.

## Supplementary information

**Additional file 1.** Sample case for calculating the CV of volumetric data from different scanners in a single subject.

**Additional file 2.** Brain volumetry quantified by different automatic segmentation tools on multiple scanners.

## Data Availability

The datasets used and/or analysed during this study are available from the corresponding author on reasonable request.

## Abbreviations

ABS: Automated brain segmentation
CV: Coefficient of variation
MRI: Magnetic resonance imaging
WM: White matter
GM: Gray matter
CSF: Cerebrospinal fluid
MMSE: Mini-mental state examination
SD: Standard deviation
