# Supplementary material for: Inter-scanner reproducibility of brain volumetry: influence of automated brain segmentation software
Source: BMC Neurosci. 2020 Sep 4;21:35. doi: 10.1186/s12868-020-00585-1 (PMC7472704; doi:10.1186/s12868-020-00585-1)
Supplement: Supplementary file 1 — Additional file 1. Sample case for calculating the CV of volumetric data from different scanners in a single subject. [file 12868_2020_585_MOESM1_ESM.docx]

**Inter-scanner reproducibility of brain volumetry: influence of automated brain segmentation software**

**Additional file 1.** Sample case for calculating the CV of volumetric data from different scanners in a single subject

| Region | Freesurfer | | | | FSL-FIRST | | | | AccuBrain | | | |
| --- | --- | --- | --- | --- | --- | --- | --- | --- | --- | --- | --- | --- |
|  | GE | SIEMENS | PHILIPS | **CV** | GE | SIEMENS | PHILIPS | **CV** | GE | SIEMENS | PHILIPS | **CV** |
| WM | 29.49 | 30.65 | 36.02 | 0.109 | N.A. | N.A. | N.A. | N.A. | 31.10 | 31.00 | 31.30 | 0.005 |
| GM | 46.97 | 47.66 | 54.46 | 0.083 | N.A. | N.A. | N.A. | N.A. | 45.00 | 43.10 | 43.80 | 0.022 |
| Hippocampus L | 0.334 | 0.374 | 0.407 | 0.098* | 0.293 | 0.255 | 0.229 | 0.124 | 0.232 | 0.262 | 0.264 | 0.071 |
| Hippocampus R | 0.322 | 0.336 | 0.391 | 0.104 | 0.298 | 0.287 | 0.271 | 0.048 | 0.215 | 0.246 | 0.243 | 0.073 |
| Amygdala L | 0.129 | 0.156 | 0.164 | 0.123 | 0.104 | 0.067 | 0.089 | 0.215 | 0.127 | 0.127 | 0.124 | 0.014 |
| Amygdala R | 0.155 | 0.140 | 0.173 | 0.106 | 0.109 | 0.043 | 0.101 | 0.427 | 0.161 | 0.147 | 0.152 | 0.046 |
| LatVent L | 0.197 | 0.173 | 0.228 | 0.138 | N.A. | N.A. | N.A. | N.A. | 0.37 | 0.327 | 0.344 | 0.062 |
| LatVent R | 0.299 | 0.297 | 0.346 | 0.088 | N.A. | N.A. | N.A. | N.A. | 0.438 | 0.411 | 0.425 | 0.032 |
| VentralDC L | 0.255 | 0.289 | 0.341 | 0.147 | N.A. | N.A. | N.A. | N.A. | 0.155 | 0.156 | 0.16 | 0.017 |
| VentralDC R | 0.234 | 0.274 | 0.332 | 0.176 | N.A. | N.A. | N.A. | N.A. | 0.164 | 0.168 | 0.178 | 0.042 |
| Thalamus L | 0.611 | 0.615 | 0.687 | 0.067 | 0.655 | 0.476 | 0.560 | 0.159 | 0.516 | 0.53 | 0.509 | 0.021 |
| Thalamus R | 0.493 | 0.553 | 0.633 | 0.125 | 0.577 | 0.450 | 0.538 | 0.125 | 0.461 | 0.499 | 0.471 | 0.041 |
| Caudate L | 0.245 | 0.228 | 0.289 | 0.124 | 0.210 | 0.174 | 0.237 | 0.153 | 0.156 | 0.182 | 0.175 | 0.079 |
| Caudate R | 0.217 | 0.233 | 0.278 | 0.130 | 0.249 | 0.162 | 0.201 | 0.214 | 0.189 | 0.198 | 0.191 | 0.025 |
| Putamen L | 0.456 | 0.446 | 0.560 | 0.130 | 0.345 | 0.331 | 0.355 | 0.035 | 0.352 | 0.376 | 0.384 | 0.045 |
| Putamen R | 0.451 | 0.468 | 0.551 | 0.109 | 0.361 | 0.339 | 0.373 | 0.048 | 0.35 | 0.383 | 0.372 | 0.046 |
| Pallidum L | 0.101 | 0.101 | 0.154 | 0.258 | 0.128 | 0.121 | 0.114 | 0.058 | 0.099 | 0.102 | 0.111 | 0.059 |
| Pallidum R | 0.108 | 0.110 | 0.140 | 0.150 | 0.133 | 0.131 | 0.116 | 0.073 | 0.106 | 0.100 | 0.104 | 0.031 |
| Accumbens L | 0.046 | 0.067 | 0.058 | 0.185 | 0.027 | 0.036 | 0.014 | 0.431 | 0.029 | 0.036 | 0.035 | 0.112 |
| Accumbens R | 0.048 | 0.061 | 0.058 | 0.122 | 0.026 | 0.032 | 0.025 | 0.137 | 0.036 | 0.036 | 0.038 | 0.024 |

For a specific subject, the CV value was calculated within the volumetric data from a specific quantification tool. *Taking WM volume measured by Freesurfer for example, CV was calculated as the SD of WM volume ratios (0.037) divided by the mean of volume ratios (0.372), resulting in 0.098. Similarly, it was calculated for FSL-FIRST results (0.124) and AccuBrain results (0.071). In this way, the CV was calculated for each subject and each quantification tool, and then these CV values were measured with mean and SD among the study cohort as shown in Table 2. GM=gray matter, LatVent=lateral ventricle, N.A.=Not available, VentralDC=Ventral diencephalon, WM=white matter. L, left; R, right
