# Supplementary material for: Inter-scanner reproducibility of brain volumetry: influence of automated brain segmentation software
Source: BMC Neurosci. 2020 Sep 4;21:35. doi: 10.1186/s12868-020-00585-1 (PMC7472704; doi:10.1186/s12868-020-00585-1)
Supplement: Supplementary file 2 — Additional file 2. Brain volumetry quantified by different automatic segmentation tools on multiple scanners. [file 12868_2020_585_MOESM2_ESM.docx]

**Inter-scanner reproducibility of brain volumetry: influence of automated brain segmentation software**

**Additional file 2.** Brain volumetry quantified by different automatic segmentation tools on multiple scanners.

| Region | FreeSurfer | | | FSL-FIRST | | | AccuBrain | | |
| --- | --- | --- | --- | --- | --- | --- | --- | --- | --- |
|  | GE | SIEMENS | PHILIPS | GE | SIEMENS | PHILIPS | GE | SIEMENS | PHILIPS |
| WM | 29.79 (1.43) | 30.12 (1.34) | 34.36 (4.81) | N.A. | N.A. | N.A. | 32.53 (0.98) | 32.24 (1.06) | 32.29 (1.03) |
| GM | 44.84 (1.95) | 43.94 (1.79) | 48.02 (6.48) | N.A. | N.A. | N.A. | 44.53 (1.15) | 43.30 (0.96) | 44.33 (2.08) |
| Hippocampus L | 0.288 (0.029) | 0.296 (0.029) | 0.322 (0.052) | 0.263 (0.022) | 0.220 (0.037) | 0.235 (0.042) | 0.208 (0.028) | 0.214 (0.034) | 0.214 (0.028) |
| Hippocampus R | 0.295 (0.023) | 0.302 (0.025) | 0.335 (0.057) | 0.276 (0.020) | 0.243 (0.033) | 0.241 (0.046) | 0.216 (0.022) | 0.223 (0.031) | 0.222 (0.027) |
| Amygdala L | 0.110 (0.009) | 0.125 (0.011) | 0.127 (0.020) | 0.093 (0.015) | 0.085 (0.018) | 0.063 (0.013) | 0.116 (0.008) | 0.122 (0.008) | 0.114 (0.008) |
| Amygdala R | 0.123 (0.014) | 0.121 (0.012) | 0.141 (0.026) | 0.088 (0.019) | 0.079 (0.018) | 0.061 (0.024) | 0.139 (0.011) | 0.138 (0.012) | 0.133 (0.011) |
| LatVent L | 0.397 (0.164) | 0.381 (0.170) | 0.435 (0.187) | N.A. | N.A. | N.A. | 0.492 (0.131) | 0.491 (0.137) | 0.482 (0.123) |
| LatVent R | 0.349 (0.121) | 0.341 (0.123) | 0.386 (0.134) | N.A. | N.A. | N.A. | 0.457 (0.096) | 0.446 (0.099) | 0.447 (0.087) |
| VentralDC L | 0.257 (0.021) | 0.265 (0.019) | 0.300 (0.048) | N.A. | N.A. | N.A. | 0.142 (0.008) | 0.153 (0.006) | 0.153 (0.007) |
| VentralDC R | 0.254 (0.017) | 0.264 (0.015) | 0.301 (0.046) | N.A. | N.A. | N.A. | 0.156 (0.008) | 0.165 (0.006) | 0.165 (0.008) |
| Thalamus L | 0.544 (0.046) | 0.533 (0.040) | 0.609 (0.103) | 0.574 (0.049) | 0.527 (0.036) | 0.491 (0.035) | 0.498 (0.023) | 0.502 (0.025) | 0.485 (0.020) |
| Thalamus R | 0.477 (0.033) | 0.495 (0.031) | 0.548 (0.086) | 0.541 (0.048) | 0.512 (0.037) | 0.472 (0.039) | 0.466 (0.024) | 0.481 (0.024) | 0.463 (0.019) |
| Caudate L | 0.249 (0.026) | 0.231 (0.024) | 0.272 (0.051) | 0.230 (0.030) | 0.210 (0.042) | 0.211 (0.042) | 0.192 (0.021) | 0.197 (0.019) | 0.201 (0.021) |
| Caudate R | 0.238 (0.025) | 0.237 (0.026) | 0.271 (0.048) | 0.233 (0.030) | 0.205 (0.045) | 0.203 (0.051) | 0.211 (0.028) | 0.212 (0.025) | 0.210 (0.026) |
| Putamen L | 0.372 (0.051) | 0.384 (0.041) | 0.418 (0.083) | 0.327 (0.027) | 0.328 (0.028) | 0.319 (0.034) | 0.335 (0.025) | 0.349 (0.029) | 0.351 (0.027) |
| Putamen R | 0.366 (0.042) | 0.365 (0.041) | 0.420 (0.077) | 0.323 (0.029) | 0.320 (0.038) | 0.323 (0.029) | 0.326 (0.029) | 0.341 (0.030) | 0.335 (0.027) |
| Pallidum L | 0.116 (0.023) | 0.107 (0.020) | 0.123 (0.024) | 0.119 (0.007) | 0.106 (0.011) | 0.112 (0.009) | 0.099 (0.006) | 0.105 (0.006) | 0.108 (0.007) |
| Pallidum R | 0.116 (0.019) | 0.105 (0.021) | 0.128 (0.020) | 0.119 (0.007) | 0.105 (0.017) | 0.113 (0.008) | 0.099 (0.006) | 0.100 (0.006) | 0.098 (0.007) |
| Accumbens L | 0.040 (0.009) | 0.040 (0.012) | 0.041 (0.035) | 0.035 (0.005) | 0.028 (0.007) | 0.031 (0.008) | 0.029 (0.003) | 0.028 (0.004) | 0.029 (0.003) |
| Accumbens R | 0.042 (0.007) | 0.040 (0.010) | 0.043 (0.026) | 0.026 (0.006) | 0.020 (0.006) | 0.021 (0.008) | 0.030 (0.003) | 0.029 (0.003) | 0.032 (0.003) |

The mean and SD of brain volume ratios (% of intracranial brain volume) from different scanners with different processing tools are provided. GM=gray matter, LatVent=lateral ventricle, N.A.=Not available, VentralDC=Ventral diencephalon, WM=white matter. L, left; R, right.
